# Longitudinal Evaluation of Sternocleidomastoid Muscle Stiffness and Self-Reported Fibrosis-Related Symptoms After Radiotherapy in Patients with Head and Neck Cancer

**DOI:** 10.3390/cancers18121928

**Published:** 2026-06-13

**Authors:** Kaat Verbeelen, An De Groef, Kaat Van Aperen, Ceren Gursen, Sandra Nuyts, Nele Devoogdt, Michel G. C. A. M. Mertens

**Affiliations:** 1 MOVANT Research Group, Department of Rehabilitation Sciences and Physiotherapy, University of Antwerp, 2610 Antwerp, Belgium; kaat.verbeelen@uantwerpen.be (K.V.); ceren.guersen@kuleuven.be (C.G.); 2 Department of Rehabilitation Sciences, University of Leuven, 3000 Leuven, Belgium; kaat.vanaperen@kuleuven.be (K.V.A.); nele.devoogdt@kuleuven.be (N.D.); 3 CarEdOn Research Group, 3000 Leuven, Belgium; 4 Pain in Motion International Research Group, 1000 Brussels, Belgium; 5 Laboratory of Experimental Radiotherapy, Department of Oncology, University of Leuven, 3000 Leuven, Belgium; sandra.nuyts@uzleuven.be; 6 Faculty of Rehabilitation Sciences, Hacettepe University, 06100 Ankara, Turkey; 7 Department of Radiation Oncology, Leuven Cancer Institute, University Hospitals Leuven, 3000 Leuven, Belgium

**Keywords:** head and neck cancer, radiotherapy, radiation fibrosis, shear wave elastography, sternocleidomastoid muscle, muscle stiffness, patient-reported outcomes

## Abstract

Background/Objectives: Post-radiation fibrosis is a common complication in patients with head and neck cancer (HNC), characterised by increased tissue stiffness and functional limitations. Shear wave elastography (SWE) enables objective assessment of tissue stiffness, while self-reported outcome measures provide insight into symptom burden. However, the longitudinal evolution of these outcomes and their relationship remains insufficiently described. This study aimed to characterise post-radiation fibrosis over time using objective SWE measurements of sternocleidomastoid (SCM) muscle stiffness and self-reported fibrosis-related symptoms, and to examine their longitudinal association. Methods: This prospective longitudinal study included 56 patients with HNC undergoing primary or postoperative (chemo)radiotherapy. Muscle stiffness was measured using SWE, and fibrosis-related symptoms were assessed with the Lymphoedema Symptom Intensity and Distress Survey-Head and Neck (LSIDS-H&N) at 1 week, 6 weeks, 12 weeks, 6 months, and 12 months after the start of radiotherapy. Longitudinal analyses were performed using mixed-effects models adjusted for surgery and radiation dose, and within-subject associations were evaluated using repeated measures correlation. Results: Muscle stiffness increased over time, with estimated mean stiffness values rising from 4.12 m/s at baseline to 4.76 m/s at 6 months and 4.92 m/s at 12 months. Significant increases were observed at 6 months (*p* = 0.007) and 12 months (*p* = 0.012) compared with baseline. In contrast, self-reported fibrosis-related symptoms remained stable, with no significant differences between time points. No significant within-subject association was found between muscle stiffness and self-reported fibrosis-related symptoms (r = −0.13, *p* = 0.498). Conclusions: Muscle stiffness measured with SWE increased over time following radiotherapy, whereas self-reported fibrosis-related symptoms remained stable. No significant longitudinal association was found between these outcomes, suggesting they may capture different aspects of fibrosis.

## Simple Summary

Patients with head and neck cancer often develop fibrosis after radiotherapy, which can lead to tissue stiffness, pain, and limitations in daily functioning. However, it remains unclear how these changes develop over time and whether objective tissue changes are reflected in patients’ symptoms. This study followed 56 patients during the first year after radiotherapy and evaluated neck muscle stiffness using ultrasound-based imaging together with self-reported fibrosis-related symptoms. Muscle stiffness gradually increased over time, particularly after 6 and 12 months, while self-reported symptoms remained relatively stable. In addition, no clear relationship was found between objective muscle stiffness and patient-reported symptoms. These findings suggest that objective imaging and self-reported outcomes may capture different aspects of radiation-induced fibrosis. Combining both approaches may improve the assessment and monitoring of fibrosis in clinical practice and support more targeted rehabilitation after treatment.

## 1. Introduction

Head and neck cancer (HNC) is the seventh most common cancer worldwide [1]. It refers to a broad category of cancers that originate from the anatomical locations that make up the upper aerodigestive tract. Major risk factors for HNC include tobacco use and chronic alcohol consumption [2,3]. Additionally, oncogenic viruses, such as human papillomavirus and Epstein–Barr virus, have been identified as significant contributors to the development of HNC [4,5].

Radiotherapy is a fundamental component of HNC treatment [6]. However, it can be associated with a range of acute and chronic side effects affecting the soft tissues and sensory functions of the head and neck region [7]. Among these, post-radiation fibrosis is particularly common, affecting more than 50% of patients within one to eight years following treatment [8].

Post-radiation fibrosis results from radiation-induced damage to soft tissue within the treated area [9,10,11]. The risk and severity of fibrosis are influenced by the radiation dose and the size of the treated area, with higher doses and larger fields associated with increased fibrotic changes [12,13]. Notably, the severity of fibrosis often progresses over time, particularly during the first one to two years following treatment [14,15]. As survival rates continue to improve, an increasing number of patients with HNC are living with long-term treatment-related sequelae. Consequently, late effects such as fibrosis have become increasingly important determinants of long-term functioning and quality of life [16]. Post-radiation fibrosis is associated with substantial morbidity, including reduced tongue mobility, cervical dystonia or neck muscle spasms, and shoulder dysfunction [8,13]. Fibrosis is also characterised by increased tissue stiffness, which reflects a key characteristic of the condition [17,18]. Increased tissue stiffness may be clinically relevant because fibrotic tissue remodelling has been associated with pain, movement dysfunction, and reduced quality of life in several clinical conditions [17,19,20]. Excessive extracellular matrix deposition and tissue remodelling can alter the mechanical properties of muscles and surrounding connective tissues, potentially impairing tissue mobility and increasing mechanical loading during movement [21,22,23]. Furthermore, fibrotic changes have been linked to inflammatory processes and sensitisation of nociceptive structures, which may contribute to the development and persistence of myofascial pain [21,22,23]. Although this relationship remains insufficiently investigated in patients with HNC, increased tissue stiffness may represent an important contributor to treatment-related symptom burden and functional impairment. In HNC, the sternocleidomastoid (SCM) muscle is a major structure within the radiotherapy field and is frequently considered a source of pain and functional limitations, such as trismus and restricted movement in the neck and shoulder, potentially due to post-radiation fibrosis, which may negatively affect quality of life [10,11,24,25]. Additionally, studies have demonstrated increased stiffness in the irradiated SCM compared to non-irradiated controls, measured with shear wave elastography (SWE) [26,27].

In addition to radiotherapy, surgical treatment in HNC may also contribute to the development of fibrosis, as tissue disruption, wound healing, and scar formation can lead to fibrotic changes in the affected region [28,29]. This is particularly relevant in cases involving extensive resection or reconstructive procedures, where postoperative scarring and soft tissue remodelling may further exacerbate stiffness and functional limitations [28].

SWE is a promising and innovative non-invasive ultrasound method that enables a detailed, objective evaluation of tissue stiffness [30,31,32]. SWE applies an acoustic radiation force to generate shear waves within the tissue: the velocity of these waves correlates positively with tissue stiffness [32]. This information is visualised through colour-coded elastograms that illustrate both the extent and severity of tissue stiffness changes and can indirectly provide more details on the severity of fibrosis.

Fibrosis can also be assessed using self-reported outcome measures, including the Lymphoedema Symptom Intensity and Distress Survey-Head and Neck (LSIDS-H&N) questionnaire [33]. The LSIDS-H&N proved to be a reliable and valid tool for assessing symptom burden and distress in patients with HNC [33]. Self-reported outcomes provide insight into patients’ symptom burden and health-related quality of life [34]. Combining objectively measured fibrosis with self-reported fibrosis-related symptoms may offer a more comprehensive understanding of the complications that arise after radiotherapy. Such an integrated approach has the potential to enhance clinical evaluation, facilitate early intervention, and guide personalised rehabilitation strategies.

Previous studies assessing radiation-induced fibrosis in HNC have predominantly focused on either objective or self-reported outcomes separately. Regarding objective assessment, SWE has been used in a limited number of studies [26,27]. Cross-sectional studies have demonstrated increased tissue stiffness in irradiated muscles compared to non-irradiated controls [26], while a prospective longitudinal study by Wen et al. in patients with nasopharyngeal carcinoma showed a progressive increase in sternocleidomastoid (SCM) muscle stiffness up to 18 months after radiotherapy, with moderate correlations with clinician-rated fibrosis severity (Late Effects Normal Tissue/Subjective Objective Management Analytic; LENT-SOMA) [27]. However, longitudinal SWE evidence in the broader HNC population remains limited.

On the self-reported side, longitudinal studies have shown that radiotherapy-related complications, such as trismus, are associated with persistent and worsening impairments in quality of life over time [35,36]. However, these studies focus on specific functional outcomes rather than self-reported fibrosis-related symptoms. Self-reported outcome measures, such as the LSIDS-H&N, have primarily been used for instrument development and validation rather than for describing the longitudinal evolution of self-reported fibrosis-related symptoms after radiotherapy [33].

Some studies have explored associations between tissue stiffness and quality-of-life outcomes, but these analyses were cross-sectional and did not assess changes over time within patients [37]. Consequently, the longitudinal evolution of both SCM stiffness and self-reported fibrosis-related symptoms remains insufficiently described, and their longitudinal association has not been investigated. Therefore, it remains unclear whether changes in SCM stiffness are reflected in self-reported fibrosis-related symptoms over time.

Therefore, the primary objective of this study was to characterise post-radiation fibrosis across multiple time points using both objective SWE measurements of SCM stiffness and self-reported fibrosis-related symptom scores (LSIDS-H&N). The secondary objective was to examine the longitudinal association between the objectively measured SCM stiffness and self-reported fibrosis-related symptoms.

It was hypothesised that post-radiation fibrosis will progress over time, as assessed by objective SWE measurements of SCM stiffness and self-reported fibrosis-related symptoms (LSIDS-H&N scores). Furthermore, there will be an association between objective muscle stiffness assessed by SWE as an indicator of fibrosis and self-reported fibrosis-related symptoms (LSIDS-H&N scores) across multiple time points.

## 2. Materials and Methods

This prospective longitudinal study was conducted as part of the EffEx-HN clinical trial, which investigates the efficacy of a comprehensive exercise programme in individuals undergoing treatment for HNC [38]. The trial is registered in ClinicalTrials.gov (NCT05256238), and ethical approval was granted by the Ethics Committee of the University Hospital Leuven (UZ Leuven; reference number S65549). Data were collected between April 2023 and August 2025. Participants were recruited from the EffEx-HN study population at the University Hospital Leuven and were invited to participate in this longitudinal assessment study. All participants provided written informed consent in accordance with the Declaration of Helsinki [39].

### 2.1. Participants

Participants were eligible if they were 18 years or older and diagnosed with a primary malignant tumour in the head and neck region (oral cavity, nasal cavity and sinuses, pharynx, larynx, salivary glands, or thyroid). All participants were scheduled for curative primary or postoperative (chemo)radiotherapy and had an Eastern Cooperative Oncology Group (ECOG) performance status of 0 or 1. Additional inclusion criteria included the ability to complete baseline assessments within one week after the start of radiotherapy and the physical and mental capacity to participate in a supervised exercise intervention. Exclusion criteria included palliative treatment or the presence of distant metastases. For the purpose of this specific study on the characterisation of post-radiation fibrosis across multiple time points, only patients in whom the SCM muscle region (unilateral or bilateral) was included in the radiotherapy fields were eligible. Otherwise, they were excluded from this analysis. For both aims of the present study, the control and intervention groups of the EffEx study were combined, as no differences were found between groups for the primary outcomes, including health-related quality of life and physical functioning. No formal sample size calculation was performed for this secondary longitudinal analysis. The sample size was determined pragmatically based on participant inclusion during the period in which the SWE device was available at the study site.

### 2.2. Procedure

All assessments were conducted by a single assessor (KVA), who holds a Master of Science in Physiotherapy and Rehabilitation Sciences and has two years of experience in the field of cancer-related lymphoedema. No blinding procedures were implemented in the present longitudinal analysis.

Measurements were performed at five predefined time points: 1 week (T0), 6 weeks (T1), 12 weeks (T2), 6 months (T3), and 12 months (T4) after the start of radiotherapy. At each time point, participants completed self-reported outcome measures and clinical assessments. SWE measurements were conducted during regular study visits based on the availability of the SWE device and participants’ willingness to undergo the additional measurement. The additional SWE assessment extended the regular study visit by approximately 15 min. As a result, SWE data are incomplete and not uniformly distributed across time points. A participant was included in the analysis if assessments were performed on at least 2 of the 5 predefined timepoints. The LSIDS-H&N was completed at every time point at which the patient was assessed.

All measurements were performed at the Department of Physical Medicine and Rehabilitation of the University Hospital Leuven.

### 2.3. Participant Characteristics

Participant demographic and clinical characteristics were recorded in the REDCap database using data extracted from electronic medical records. Collected demographic variables included age, gender, body mass index, and genetic ancestry (categorised as White, Black, African American, or Other), marital status, and work status.

Tumour-related variables included the primary tumour location (left, right, or midline) and cancer stage according to the TNM classification [40]. Treatment-related variables comprised treatment modality (surgery and chemo(radiotherapy) or only chemo(radiotherapy)), type of surgery, neck dissection and radiation side of the SCM. The radiation dose at the level of the SCM on each side of the neck (elective, or high) was also registered.

### 2.4. Outcome Measures

#### 2.4.1. Muscle Stiffness

Muscle stiffness was measured using SWE with the Aixplorer ultrasound system (SuperSonic Imagine, Aix-en-Provence, France), equipped with an L18-5 linear transducer. The musculoskeletal preset was used, and the transducer was aligned longitudinally along the SCM. The imaging depth was set to 3.0 cm, and the elasticity scale was fixed at 180 kPa. The region of interest box was tailored in height to fit the muscle’s anatomical contours, with a standardised length of 3 cm on all images.

**Figure 1:**
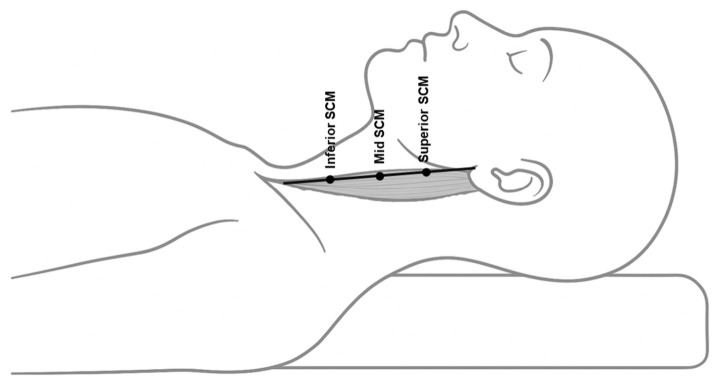
Overview of the measuring points on the SCM.

Participants were assessed in a supine position on a treatment table, with their head in a neutral alignment, arms and legs relaxed, and a roll placed under the knees for comfort. The SCM was mapped using a tape measure from the earlobe to the sternoclavicular joint. Three specific points were marked at 25% (MP1—superior SCM), 50% (MP2—mid SCM), and 75% (MP3—inferior SCM) along this line (see Figure 1) to standardise measurement locations.

At each marked site, two 15-s SWE videos were collected, resulting in 6 recordings per side and a total of 12 per participant if the patient received radiotherapy on both sides and still had an SCM.

The acquired SWE data were analysed using ElastoGUI, an open-source tool developed in Matlab^®^ (https://bio.tools/elastogui, accessed on 18 February 2024), MATLAB R2024b (MathWorks, Natick, MA, USA). For each elastography video, a rectangular region of interest was manually delineated within the anatomical borders of the SCM on the colour-coded stiffness map. This ensured that stiffness measurements were restricted to the SCM and did not include surrounding tissues. Shear wave velocity (SWV) values, expressed in meters per second (m/s), were extracted from the elastography colour maps calculated with the ElastoGUI tool. For each video recording, the ten frames with the lowest standard deviation were identified, and their mean value was computed. The final stiffness value was then determined by averaging the results obtained from the two recordings [41]. Higher SWV values indicate greater tissue stiffness [32].

#### 2.4.2. Self-Reported Fibrosis-Related Symptoms

The LSIDS-H&N v2.0 is a valid questionnaire designed to measure the impact of lymphoedema and fibrosis in patients with HNC. It assesses both symptom burden and functional limitations [33,42].

The instrument consists of 31 items grouped into seven clusters: Soft Tissue Sensation (9 items), Activity (6 items), Oral Function (5 items), Resources (2 items), Neurological Sensation (2 items), Biobehavioural (4 items), and Sexuality (3 items) [42]. Each item is first scored dichotomously (“yes” or “no”). A response of “no” receives a score of 0. If the respondent answers “yes,” they also rate the intensity and distress associated with the symptom, each on a 5-point scale (1 = slight, 5 = severe). The item score is calculated as the sum of the intensity and distress ratings, resulting in a possible range of 0 to 10 for each cluster.

The overall LSIDS-H&N score is calculated by averaging all 31 items, resulting in a total score ranging from 0 to 10. Up to 5 missing items are permitted; if more than 5 items are missing, the overall LSIDS-H&N score is not calculated and is considered missing. Cluster scores, calculated as the mean of the item scores within a given cluster, are likewise expressed on a scale from 0 to 10. For clusters with more than two items, up to 40% missing responses are allowed. Higher scores indicate greater symptom severity and distress. In this study, the overall score of the LSIDS-H&N will be used. In the appendix (Appendix A), the analyses of the cluster scores are available.

### 2.5. Statistical Analysis

Continuous variables are presented as means with standard deviations (SD), while categorical variables are reported as frequencies and percentages. For muscle stiffness measured with SWE, the mean of the three measurement points per side was calculated, as previous work demonstrated greater reliability for averaged values.

To evaluate the longitudinal evolution of muscle stiffness measured with SWE (or shear wave velocity in m/s), linear mixed-effects models were used when the data were approximately normally distributed. If the normality assumption was not met, generalised linear mixed models with a gamma distribution were applied, as shear wave velocity is a continuous variable that may be skewed. For generalised linear mixed models with a log link, results were exponentiated and presented as mean ratios, indicating the relative change in the outcome between time points. A mean ratio below 1 indicates a decrease, whereas a value above 1 indicates an increase. For each participant, side(s) of the SCM that received radiotherapy were treated as separate data points in the analysis.

For self-reported fibrosis-related symptoms assessed using the LSIDS-H&N overall score, linear mixed models were used when the outcome was approximately normally distributed. If this assumption was not met, generalised linear mixed models were applied, with the distribution selected based on the characteristics of the data and overall model fit.

Normality was assessed using the Shapiro–Wilk test in combination with visual inspection of histograms and Q–Q plots. Model assumptions were further evaluated using residual-versus-fitted plots to assess homoscedasticity and potential model misspecification.

All mixed-effects models accounted for repeated measurements over time (within 1 week, 6 weeks, 12 weeks, 6 months, and 12 months after the start of radiotherapy). Mixed models can accommodate missing data under the missing-at-random assumption. To account for the dependence of observations within individuals, a random intercept for each participant was included. These models can also include participants with only a single observation, as they estimate both population-level and subject-specific effects.

Radiation dose and surgery were included as covariates due to their known impact on the development and progression of post-radiation fibrosis [13,28,29]. For SWE analyses, radiation dose was considered separately for each data point, corresponding to the specific SCM muscle measured. For the SWE analyses, radiation dose was determined separately for each data point, corresponding to the specific SCM muscle being assessed. Two radiation dose levels were defined for each SCM: elective dose and high dose. Elective dose referred to SCM regions receiving 50–54 Gy, whereas high dose referred to SCM regions receiving 60–70 Gy directed at the primary tumour or involved lymph nodes. Dose classification was based on the patients’ radiation therapy reports and verified using MRI images to determine whether the irradiated region was located near the SCM muscle. In addition, it was recorded whether surgery had been performed on the same side as the SWE measurement. Surgical procedures were only considered relevant when they involved tumour resection in the region of the SCM muscle or a neck dissection.

For the LSIDS-H&N analyses, a single radiation dose value per participant was used, defined as the highest dose received by either SCM muscle. Surgery was included as a binary variable (yes/no) at the patient level. Post hoc comparisons were performed using Tukey’s method when appropriate.

To assess the within-subject association over time between muscle stiffness measured by SWE and self-reported fibrosis-related symptoms assessed with the LSIDS-H&N, repeated measures correlation was used, thereby accounting for non-independence of observations within individuals [43,44,45]. This method requires at least two observations per participant; therefore, only participants with ≥2 paired measurements were included in this analysis. For this analysis, a single side per participant was selected to align with the participant-level LSIDS-H&N outcome. The side receiving the highest radiation dose was chosen. If both sides received the same radiation dose, one side was selected using computer-based randomisation. Surgery was not included in this analysis, as the SCM side selected for analysis was not always the same side on which surgery had been performed. Additionally, only paired observations in which both SWE and LSIDS-H&N data were available were included. The LSIDS-H&N overall score was considered missing when more than five items were unanswered, in accordance with its scoring guidelines. As a result, not all measurement time points were included in this analysis, even when the questionnaire had been completed, because no score was available.

Correlation coefficients were interpreted as very strong (r > 0.8), strong (0.6–0.8), moderate (0.3–0.6), or weak (r < 0.3) [46]. Statistical significance was set at α < 0.05.

All statistical analyses were conducted in R (version 4.5.2). Mixed-effects models were fitted using the add-on packages lme4 [47], pbkrtest [48], and emmeans [49]. Longitudinal correlations were assessed with the rmcorr package [50].

## 3. Results

### 3.1. Patient Characteristics

A total of 56 patients were included in the analysis. Patient and treatment characteristics are presented in Table 1.

An overview of missing data by time point is provided in Table 2. Not all participants provided data at every time point, resulting in an unbalanced dataset across follow-up measurements. Missing SWE data occurred when ultrasound equipment was unavailable or when participants declined measurement. Missing LSIDS-H&N total scores were primarily due to the instrument’s scoring guidelines. Differences in the number of observations between SWE and LSIDS-H&N reflect both distinct mechanisms of missing data and the different levels of measurement (SCM side vs. participant).

### 3.2. Longitudinal Evolution

For the longitudinal evolution of both muscle stiffness measured with SWE (SWV in m/s) and self-reported fibrosis-related symptoms assessed using the LSIDS-H&N overall score, the normality assumption was not met based on the Shapiro–Wilk test and visual inspection of histograms and Q–Q plots. Therefore, generalised linear mixed models with gamma and negative binomial distributions were applied. Residual-versus-fitted plots showed no major violations of model assumptions, including homoscedasticity and absence of systematic patterns in the residuals.

**Figure 2:**
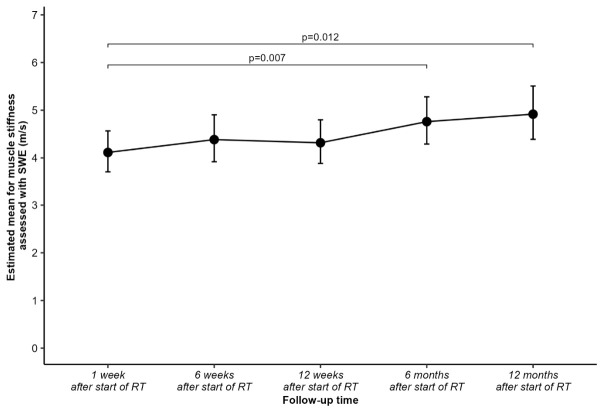
Estimated marginal means of muscle stiffness (shear wave velocity, m/s) across follow-up time points after the start of radiotherapy. Error bars represent 95% confidence intervals derived from the mixed-effects model.

Muscle stiffness showed a descriptive increase over time (Table 3). Mixed-effects modelling demonstrated a significant overall effect of time (*p* = 0.002; Table 4; Figure 2). Post hoc analyses indicated that muscle stiffness was significantly higher at 6 months (ratio = 0.86; *p* = 0.007) and 12 months (ratio = 0.84; *p* = 0.012) compared with baseline, while no other pairwise differences were observed (Table 4). These ratios indicate that muscle stiffness was approximately 14% and 16% higher at 6 and 12 months, respectively, compared with baseline.

**Figure 3:**
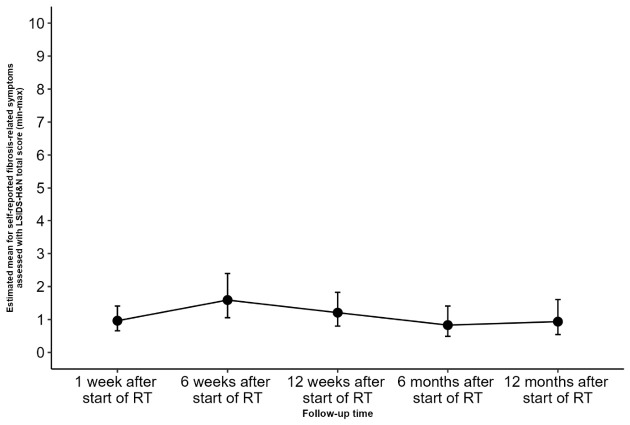
Estimated marginal means of self-reported fibrosis-related symptoms (LSIDS-H&N score) across follow-up time points after the start of radiotherapy. Error bars represent 95% confidence intervals derived from the mixed-effects model.

Self-reported fibrosis-related symptoms remained descriptively stable over time (Table 3), with no significant overall effect of time observed in the mixed-effects model (*p* > 0.05; Table 4; Figure 3). Analyses of the individual LSIDS-H&N clusters are provided in the Appendix. Negative binomial mixed-effects models showed significant longitudinal effects of time for activity, oral, and sexuality outcomes, with changes primarily driven by increases at T1 in activity, oral, biobehavioral, and sexuality domains, alongside decreases in neurological sensation domains.

### 3.3. Longitudinal Association

The within-subject association over time between muscle stiffness measured with SWE and self-reported fibrosis-related symptoms assessed with the LSIDS-H&N showed no significant correlation (r = −0.13; 95% CI −0.48 to 0.25; *p* = 0.498), based on 52 observations from 24 participants.

## 4. Discussion

This study examined the longitudinal evolution of muscle stiffness, measured with SWE, and self-reported fibrosis-related symptoms, assessed with the LSIDS-H&N, in patients with HNC following radiotherapy. Importantly, to our knowledge, this is the first longitudinal study to investigate these outcomes simultaneously using repeated measures. The findings demonstrate a significant increase in muscle stiffness over time, whereas self-reported fibrosis-related symptoms remained stable, with no significant within-subject association between the two outcomes. This positions the current work as an early contribution in the emerging field of quantitative assessment of radiation-induced fibrosis in HNC.

### 4.1. Longitudinal Evolution of Muscle Stiffness

Muscle stiffness increased over time, with significantly higher stiffness of the SCM muscle at 6 and 12 months compared with baseline. Specifically, stiffness increased by approximately 14% at 6 months and 16% at 12 months relative to baseline values. In absolute terms, estimated mean stiffness increased from 4.12 m/s at baseline to 4.76 m/s at 6 months and 4.92 m/s at 12 months. As no significant differences were observed at earlier time points, this suggests that measurable tissue changes become apparent in the mid- to long-term period after radiotherapy rather than immediately.

The increase in stiffness (approximately 0.6–0.8 m/s) was greater than the SEM values obtained in the reliability analyses, indicating a potentially meaningful change beyond measurement variability. In addition, stiffness values in this study are clearly higher than those reported in healthy individuals (approximately 2.75 m/s) [51]. This difference is consistent with other previous SWE studies demonstrating higher stiffness in irradiated muscles compared to non-irradiated tissue in patients with HNC [37,52].

These findings are consistent with the biological understanding that radiation-induced fibrosis is a progressive process involving tissue remodelling and increasing stiffness over time [53]. The continued increase at 12 months suggests that these changes persist beyond the initial recovery phase. This is further supported by longitudinal evidence showing increased stiffness up to 18 months after radiotherapy [27]. Similar progressive patterns have also been observed in other patient populations, such as breast cancer populations, in which fibrosis develops over time [54]. In addition, surgical treatment may further contribute to fibrotic tissue changes through wound healing and scar formation, highlighting the multifactorial nature of fibrosis development in this population [28,29].

### 4.2. Longitudinal Evolution of Self-Reported Fibrosis-Related Symptoms

In contrast, self-reported fibrosis-related symptoms did not change significantly over time. Although mean scores increased slightly at 6 weeks and then decreased, these changes were not statistically significant, indicating no clear longitudinal evolution within the first year after radiotherapy.

The LSIDS-H&N was selected because it is intended to assess symptoms related to both lymphoedema and fibrosis in patients with HNC, thereby reflecting a broader underlying construct that encompasses multiple symptom domains [42]. However, overall LSIDS-H&N scores remained low in this sample, suggesting a relatively low level of self-reported fibrosis-related symptoms. This limited variability may have reduced the ability to detect changes over time. In addition, the broad construct of the LSIDS-H&N, encompassing a range of symptoms beyond fibrosis alone, may have limited its sensitivity to detect changes in fibrosis-specific symptoms.

This raises the possibility that more fibrosis-specific self-reported outcome measures may be more sensitive to detect changes over time. For example, the Neck Fibrosis Scale has been developed to assess fibrosis-related symptoms in patients with HNC, although its validity is limited [55].

Furthermore, although no significant changes were observed in the overall LSIDS-H&N score, outcomes of specific domains changed over time, such as activity, oral function, neurological sensations, and sexuality. This suggests that domain-specific outcomes may provide additional insight into changes over time beyond the overall score, as combining multiple symptoms into a single score may make changes in specific domains less apparent. However, it remains unclear whether these domain-specific changes are associated with objective fibrosis, as measured by muscle stiffness, or reflect other treatment-related factors. Individual factors such as symptom perception, adaptation, and coping may also influence how symptoms are reported over time [46].

### 4.3. Association Between Muscle Stiffness and Self-Reported Symptoms

No significant association was found between muscle stiffness and self-reported fibrosis-related symptoms over time. This indicates that increased stiffness does not directly translate into higher symptom burden as measured with the LSIDS-H&N.

This finding likely reflects differences between objective and subjective constructs. Muscle stiffness assessed with SWE represents a physical tissue property, whereas the LSIDS-H&N captures a broader range of physical and psychosocial dimensions. As a result, the instrument may be insufficiently sensitive to detect specific physical impairments such as reduced mobility or localised discomfort associated with increased muscle stiffness, as a measure of fibrosis. In addition, self-reported outcome measures primarily reflect perceived symptom burden and quality of life rather than direct tissue alterations, which may limit their ability to capture early fibrosis-related changes [56,57,58].

The relatively low symptom scores observed in this cohort may have further limited the ability to detect associations, as restricted variability reduces statistical power for correlation analyses. This finding is consistent with previous studies, in which both median values and interquartile ranges of LSIDS-H&N scores were similarly located in the lower half of the scale [42]. Such relatively low scores may reflect selection bias, with the study population potentially representing individuals with a milder symptom burden. Additionally, the LSIDS-H&N may not fully capture the specific pattern of complaints relevant to fibrosis-related changes in this population [59].

The absence of an association may also reflect differences in the constructs being assessed. SWE provides a localised measurement of stiffness within a single muscle, whereas the LSIDS-H&N captures a broad range of symptoms and their impact on daily functioning. Consequently, the LSIDS-H&N reflects a more global patient experience that is influenced by multiple treatment-related factors beyond the stiffness of a single muscle. This is consistent with previous research showing that objective measures and patient-reported outcomes often demonstrate only modest associations because they capture related but distinct dimensions of health and functioning [60,61,62]. More specifically, these findings align with previous research showing inconsistent relationships between objective measures of muscle stiffness and subjective symptom reports [37]. Studies using techniques such as SWE and myotonometry have demonstrated that increases in tissue stiffness do not necessarily correspond to perceived pain or stiffness across various populations, including delayed-onset muscle soreness, osteoarthritis, and chronic neck pain [63,64,65,66]. Similarly, studies comparing SWE with clinical fibrosis grading have shown that objective increases in stiffness can occur in patients who are still classified as having no clinically detectable fibrosis [19], suggesting that objective measures and subjective or clinical assessments capture different aspects of the condition rather than a single underlying construct.

Future research should therefore explore outcome measures more directly related to the physical construct of muscle stiffness, such as cervical range of motion and pain intensity, which reflect impairments at the level of body function, as well as instruments such as the Neck Disability Index, which capture limitations at the level of activities and participation [27,37,67]. In addition, more fibrosis-specific self-reported outcome measures such as the Neck Fibrosis Scale may provide a more targeted assessment of fibrosis-related symptoms. Considering these different levels may provide a broader understanding of how radiation-induced changes manifest clinically and allow for more targeted evaluation and intervention at each level of the International Classification of Functioning, Disability and Health framework. Such alignment of constructs may help to better understand whether objective tissue changes are reflected in more specific functional outcomes. If validated, these tools could provide more accessible alternatives to advanced imaging techniques such as SWE in routine clinical practice.

### 4.4. Clinical Implications

Self-reported outcome measures reflect symptom burden, but may not capture specific physical changes such as increased tissue stiffness. Therefore, relying on self-reported outcome measures alone may not be sufficient to assess fibrosis-related changes. Measuring muscle stiffness may add value by detecting tissue changes that are not yet reflected in symptoms. However, since the clinical relevance of these changes remains unclear, stiffness alone should at this moment not guide treatment decisions.

Combining objective, self-reported, and functional measures is important to better understand which changes are relevant for the patient and to support more targeted clinical decision-making.

### 4.5. Future Research

Future research should focus on clarifying the clinical relevance of increased muscle stiffness and its relationship with outcomes at different levels of the International Classification of Functioning, Disability and Health framework, including body function (e.g., cervical range of motion, pain intensity) and activity/participation (e.g., Neck Disability Index). In addition, longer follow-up periods are needed to better understand the temporal relationship between tissue stiffness and symptoms.

Further studies should also consider assessing additional anatomical structures, such as the suprahyoid muscles or subcutaneous tissues, to provide a more comprehensive evaluation of radiation-induced fibrosis. Finally, standardisation of SWE measurement protocols and assessment of reliability are essential to improve the robustness and reproducibility of future research.

### 4.6. Strengths and Limitations

When interpreting the results of this study, some limitations should be considered. First, missing data occurred due to the unavailability of the ultrasound device, particularly at later follow-up time points, which may have influenced the longitudinal analyses. Missing SWE measurements may have reduced the precision of the estimated stiffness trajectories and limited the ability to fully characterise the progression of SCM stiffness over time. Consequently, the magnitude of the observed increases in stiffness should be interpreted with caution, particularly at later follow-up time points where fewer observations were available. A substantial proportion of missing data was also observed at the item level of the LSIDS-H&N, which, in several cases, precluded the calculation of total scores due to predefined limits on allowable missing responses. This may be related to the questionnaire structure, as previous research has shown that certain items, particularly in sensitive domains such as sexuality, are more often left unanswered and that the questionnaire may be perceived as burdensome [42]. Consequently, missing responses may not necessarily reflect absence of symptoms. The resulting reduction in available LSIDS-H&N scores and paired SWE-LSIDS-H&N observations may have limited the ability to detect longitudinal changes in self-reported symptoms and associations between objective and self-reported outcomes. In addition, no measurement was performed prior to the start of radiotherapy, limiting the ability to distinguish treatment-induced changes from baseline variability. A further limitation is the relatively small sample size, which may have reduced statistical power and limited the ability to detect statistically significant associations. This limitation is compounded by the use of conservative statistical approaches, which, although robust to violations of model assumptions, may be less sensitive to detecting true effects in smaller samples.

Radiation dose and surgery were included as covariates to adjust for their potential confounding influence on fibrosis-related outcomes. However, the study was not designed to assess their independent effects, and their individual contributions could not be disentangled.

Although the LSIDS-H&N questionnaire was completed at each available time point, the overall score could not be calculated for all participants due to missing items, further reducing the available data for analysis.

Despite these limitations, this study has several important strengths. To our knowledge, this is the first study to longitudinally investigate muscle stiffness and self-reported fibrosis-related symptoms using repeated measures. This longitudinal design allows evaluation of changes over time in individual patients, providing valuable insight into the development of fibrosis after radiotherapy.

Furthermore, the combination of objective (SWE) and self-reported assessment (LSIDS-H&N) offers a more comprehensive understanding of treatment-related changes. While SWE is not yet widely implemented in clinical practice, this study contributes to the exploration of objective tools to assess fibrosis-related tissue changes and highlights potential directions for future clinical assessment.

Finally, all measurements were performed by a single assessor, which ensured consistency in data collection. Given that SWE is a highly operator-dependent technique, this standardisation strengthens the internal validity of the measurements.

## 5. Conclusions

After (chemo)radiotherapy for HNC, either primary or postoperative, SCM muscle stiffness measured with SWE increased over time, whereas self-reported fibrosis-related symptoms did not change. Finally, no significant within-subject association was found between muscle stiffness and self-reported fibrosis-related symptoms. These findings suggest that objective and self-reported measures capture different aspects of post-radiation fibrosis.

## Figures and Tables

**Table 1:** Patient Characteristics (*n* = 56).

Characteristic		Mean (SD)
Age (years)		64.3 (11.3)
BMI (kg/m^2^)		26.4 (5.5)
		**N (%)**
Gender		
	Female	15 (26.8%)
	Male	41 (73.2%)
Genetic ancestry		
	Black or African American	2 (3.6%)
	Other	3 (5.4%)
	White	51 (91.1%)
Marital status		
	Married/living together	40 (71.4%)
	Single/divorced/widowed	16 (28.6%)
Work status		
	Paid employed	12 (21.4%)
	Self-employed	6 (10.7%)
	Temporarily disabled for work/other	4 (7.1%)
	Unemployed/household/retired	34 (60.7%)
Tumour location		
	Hypopharynx	7 (12.5%)
	Larynx	6 (10.7%)
	Nasal cavity/paranasal sinuses	3 (5.4%)
	Nasopharynx	3 (5.4%)
	Oral cavity	11 (19.6%)
	Oropharynx	13 (23.2%)
	Other	8 (14.3%)
	Salivary gland	1 (1.8%)
	Thyroid	4 (7.1%)
Stage		
	1	9 (16.1%)
	2	10 (17.9%)
	3	16 (28.6%)
	4	21 (37.5%)
T stage		
	T0 (Unknown)	7 (12.5%)
	T1	7 (12.5%)
	T2	22 (39.3%)
	T3	10 (17.9%)
	T4	10 (17.9%)
N stage		
	N0	15 (26.8%)
	N1	21 (37.5%)
	N2	16 (28.6%)
	N3	4 (7.1%)
Treatment		
	Chemoradiotherapy	16 (28.6%)
	Radiotherapy	19 (33.9%)
	Surgery and post-operative chemoradiotherapy	10 (17.9%)
	Surgery and post-operative radiotherapy	11 (19.6%)
Surgery		
	Not applicable	35 (62.5%)
	Only neck dissection	2 (3.6%)
	Only tumour removal	1 (1.8%)
	Tumour removal and neck dissection	18 (32.1%)
Neck dissection		
	Bilateral	6 (10.7%)
	Unilateral	14 (25.0%)
	Not applicable	36 (64.3%)
Radiation side of the SCM		
	Left	6 (10.7%)
	Bilateral	46 (82.1%)
	Right	4 (7.1%)
**Radiation dose received at the SCM region per side (n = 102)**		
Elective		63
High		39

BMI = body mass index; SCM = sternocleidomastoid muscle; SD = standard deviation; T stage = primary tumour stage according to the TNM classification; N stage = regional lymph node stage according to the TNM classification.

**Table 2:** Overview of missing data by timepoint.

Timepoint	Participants Assessed for LSIDS-H&N (*n*)	LSIDS-H&N Total Score Missing * (*n*)	SWE Missing † (SCM Side Level, *n* = 102)
1 week after the start of RT	56	21	51
6 weeks after the start of RT	52	30	66
12 weeks after the start of RT	47	23	57
6 months after the start of RT	50	31	54
12 months after the start of RT	44	28	60

* LSIDS-H&N total scores were considered missing when more than five items were unanswered according to the scoring guidelines. † SWE data are reported at the SCM side level; each participant could contribute one or two sides depending on whether radiotherapy was applied unilaterally or bilaterally. Therefore, SWE counts are not directly comparable with LSIDS-H&N counts. LSIDS-H&N = Lymphoedema Symptom Intensity and Distress Survey-Head and Neck; N = Number; RT = Radiotherapy; SWE = Shear wave elastography.

**Table 3:** Descriptive statistics observed data for muscle stiffness and self-reported fibrosis-related symptoms across follow-up time points.

	Observed Data				
	1 Week After the Start of RT (Baseline)	6 Weeks After the Start of RT	12 Weeks After the Start of RT	6 Months After the Start of RT	12 Months After the Start of RT
**Muscle stiffness measured with SWE (m/s)**					
Mean (SD; range) (m/s)	3.47 (0.63; 2.26–5.24)	3.67 (0.74; 2.32–5.05)	3.83 (1.03; 3.52–4.14)	4.22 (1.86; 2.21–11.11)	5.53 (2.58; 2.03–13.31)
95% CI	3.29–3.65	3.42–3.91	3.52–4.14	3.68–4.76	4.73–6.34
N	51	36	45	48	42
**Self-reported fibrosis-related symptoms measured with LSIDS-H&N (0–10)**					
Mean (SD; range)	1.18 (1.13; 0–3.62)	1.61 (1.46; 0–4.48)	1.47 (1.42; 0–4.19)	0.95 (1.34; 0–5.74)	1.11 (1.00; 0–3.12)
95% CI	0.79–1.57	0.96–2.25	0.87–2.07	0.31–1.60	0.58–1.64
N	35	22	24	19	16

LSIDS-H&N = Lymphoedema Symptom Intensity and Distress Survey-Head and Neck; RT = Radiotherapy; SWE = Shear wave elastography.

**Table 4:** Muscle stiffness (SWE) and self-reported fibrosis-related symptoms (LSIDS-H&N) over time following radiotherapy, with estimated means derived from mixed-effects models.

	Fixed Effects				
	1 Week After the Start of RT (Baseline)	6 Weeks After the Start of RT	12 Weeks After the Start of RT	6 Months After the Start of RT	12 Months After the Start of RT
Muscle stiffness measured with SWE (m/s) *					
Estimate mean (m/s)	4.12	4.38	4.32	4.76	4.92
SE	0.22	0.25	0.23	0.25	0.28
95% CI	3.71–4.57	3.92–4.90	3.89–4.80	4.29–5.28	4.39–5.51
Tukey post hoc	Baseline < 6 months (ratio = 0.86; *p* = 0.007) and 12 months (ratio = 0.84; *p* = 0.012)				
Self-reported fibrosis-related symptoms measured with LSIDS-H&N *					
Estimated mean	0.96	1.59	1.21	0.83	0.94
SE	0.19	0.33	0.25	0.22	0.26
95% CI	0.66–1.41	1.06–2.39	0.80–1.82	0.49–1.41	0.55–1.60
Tukey post hoc	No significant pairwise differences				

* Overall effect of time: *p* = 0.002 for SWE; *p* > 0.05 for LSIDS (adjusted for radiation dose and surgery). CI = Confidence interval; LSIDS-H&N = Lymphoedema Symptom Intensity and Distress Survey-Head and Neck; RT = Radiotherapy; SWE = Shear wave elastography; SE = Standard error.

**Figure A1:**
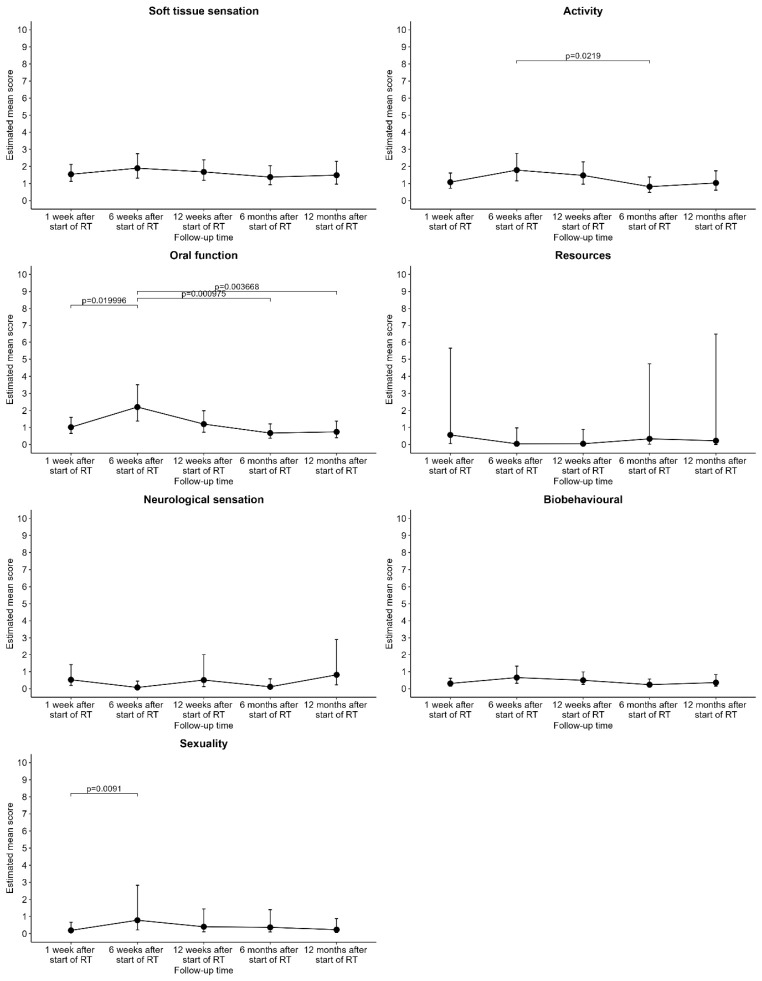
Estimated marginal mean scores for the LSIDS-H&N subdomains across follow-up time points after the start of radiotherapy.

## Data Availability

The datasets generated and/or analysed during the current study are available from the corresponding authors on reasonable request.

## Abbreviations

The following abbreviations are used in this manuscript:

HNC	Head and Neck Cancer
SWE	Shear Wave Elastography
SCM	Sternocleidomastoid Muscle
LSIDS-H&N	Lymphoedema Symptom Intensity and Distress Survey-Head and Neck
SWV	Shear Wave Velocity
RT	Radiotherapy
BMI	Body Mass Index
ECOG	Eastern Cooperative Oncology Group
TNM	Tumour, Node, Metastasis
REDCap	Research Electronic Data Capture
MRI	Magnetic Resonance Imaging
LENT-SOMA	Late Effects Normal Tissue/Subjective Objective Management Analytic
SD	Standard Deviation
SE	Standard Error
SEM	Standard Error of Measurement
CI	Confidence Interval
IR	Incidence Rate Ratio

## Appendix A

Estimated mean scores for the seven LSIDS-H&N subdomains (soft tissue sensation, activity, oral function, resources, neurological sensation, biobehavioural, and sexuality) across follow-up time points after the start of radiotherapy (RT). Data are presented as estimated marginal means with error bars representing 95% confidence intervals. Significant pairwise differences between time points are indicated with brackets and corresponding *p*-values. Higher scores indicate greater symptom burden.
